# Supplementary material for: Identification of families in need of support: Correlates of adverse childhood experiences in the right@home sustained nurse home visiting program
Source: PLoS One. 2022 Oct 3;17(10):e0275423. doi: 10.1371/journal.pone.0275423 (PMC9529103; doi:10.1371/journal.pone.0275423)
Supplement: Attachment — Submitted filename: renamed_592f5.docx [file pone.0275423.s002.docx]

**COMMENTS – Manuscript PONE-D-21-06995**

Title: “Identification of families in need of support: correlates of adverse childhood experiences in the right@home sustained nurse home visiting program”

**Important note: This review pertains only to ‘statistical aspects’ of the study and so** **‘clinical aspects’ [like medical importance,** **relevance of the study,** **‘clinical significance and implication(s)’ of the whole study, etc.] are to be evaluated [should be assessed] separately/independently. Further please note that any ‘statistical review’ is generally done under the assumption that (such) study specific methodological [as well as execution] issues are perfectly taken care of by the investigator(s). This review is not an exception to that and so does not cover clinical aspects {however, seldom comments are made only if those issues are intimately / scientifically related & intermingle with ‘statistical aspects’ of the study}. Agreed that ‘statistical methods’ are used as just tools here, however, they are vital part of methodology [and so should be given due importance].**

**COMMENTS:** Though the use of non-parametric Spearman’s ‘Correlation coefficient (ρ)’ {instead of parametric ‘Pearson’s} is appreciated, as most of measures/tools used are [though appropriate] yield data that are in ‘ordinal’ level of measurement and then application of suitable non-parametric test(s) is/are indicated/advisable even if distribution may be ‘Gaussian’ (i.e., normal)]. Moreover, most of the ‘Spearman correlation Coefficients’ reported in Table 3 [Spearman correlations of screening criteria by disclosed to MECSH nurse] are numerically very small {though sometimes significant}. In this context, please read the following:

Statistical test usually used to assess significance of Pearson’s ‘Correlation coefficient (r)’ is ‘t’ [where t = { r √ [(n-2) / (1-r^2^)] }for df=n-2, n is sample size] and here Ho is that the population/standard value of ‘r’ is zero. You need r=0.878 to be significant at 5% when n=5 but you need r=0.273 only, if n=50 & you need r=0.088 only, if n=500. Because ‘P-value’ heavily depends on sample size, it is customary to use the (available in most text books on ‘Biostatistics’ or on ‘www/net’) guidelines very strongly suggesting] to consider an absolute value of ‘Correlation coefficient for interpreting positive or negative correlations (and do not rely only on corresponding ‘P’-value but also consider an absolute value of ‘Correlation coefficient’). [This argument is equally applicable to non-parametric Spearman’s ‘Correlation coefficient (ρ)’ as well.]

This is pasted from one standard textbook on ‘Research Methodology’ and I am sure that the authors already know these things, however, it is very essential to keep the limitations in mind while interpreting results. In fact, I wonder “how even ‘Spearman’s Correlation coefficients (ρ)s’ are calculated as most of the variables are (seems to be as per lines 167-8: Spearman correlations were conducted to test the association between the presence/absence of screening factors and presence/absence of disclosed ACE risks.) ‘binary/dichotomous’ [where only biserial or point biserial correlation coefficients are appropriate”. Will you please explain? (may be correctly done, but explanation is essential).

Major part (lines 309-317: In this population, maternal smoking in pregnancy could be considered to be acting as a proxy for substance misuse, involvement with child protection services, mental health issues and family violence, suggesting that in similar populations particular consideration be given to additional and sustained support for mothers who are smoking in pregnancy. Not living with another adult was a proxy for unstable and violent relationships and major mental health issues; and anxious mood in the weeks prior to screening in pregnancy as a proxy for anxiety/depression and mental health issues during the subsequent two years. Maternal age was not a useful indicator of risk, however, receipt of state benefits was a proxy for a complex family environment) of ‘conclusion’ section is not really a [rather may/cannot be the] part of conclusion. Opinion(s) [may be even if learned from this experience], in my opinion, are not to be included in ‘conclusions’ of the study.

Overall, the presentation is confusing with respect few points {example – lines 139-140: 363 women were allocated to the trial intervention arm, and 352 commenced the intervention program what is the relevance of such grouping? No group comparison is clearly seen anywhere later. How the ‘allocation’ was performed is not given. Moreover, at few places {example - lines 167-8: Spearman correlations were conducted to test the association between the presence/absence of screening factors and presence/absence of disclosed ACE risks as we never conduct the correlations} construction of a sentence needs to be checked, I guess.

Limitations of the study are not discussed in a separate section (expected). Lines 295-97 mention only one limitation [These results cannot be generalized to population prevalence as the data were limited to those women who disclosed two or more screened-for sociodemographic factors in the context of recruitment for a sustained nurse home visiting trial.] though, which is not enough. {Does that mean {according to authors} there are none?} In my opinion, though it is a straight-forward simple study (not many methodological issues), contributes a marginal/minimal.

As pointed out in ‘important note’ above “This review pertains only to ‘statistical aspects’ of the study and so ‘clinical aspects’ should be assessed separately/independently. In my opinion, to rescue this article (which is difficult but not impossible), lot of re-vision is needed. Therefore, just short of ‘rejection’, I am recommending “Major Revision”.
