# Supplementary material for: Identification of families in need of support: Correlates of adverse childhood experiences in the right@home sustained nurse home visiting program
Source: PLoS One. 2022 Oct 3;17(10):e0275423. doi: 10.1371/journal.pone.0275423 (PMC9529103; doi:10.1371/journal.pone.0275423)
Supplement: Attachment — Submitted filename: Response to Reviewers.docx [file pone.0275423.s003.docx]

**RESPONSE TO REVIEWERS – MANUSCRIPT PONE-D-21-06995**

**Title:** Identification of families in need of support: correlates of adverse childhood experiences in the right@home sustained nurse home visiting program

| REVIEWER #1 | AUTHOR RESPONSE |
| --- | --- |
| *Important note: This review pertains only to ‘statistical aspects’ of the study and so* *‘clinical aspects’ [like medical importance,* *relevance of the study,* *‘clinical significance and implication(s)’ of the whole study, etc.] are to be evaluated [should be assessed] separately/independently. Further please note that any ‘statistical review’ is generally done under the assumption that (such) study specific methodological [as well as execution] issues are perfectly taken care of by the investigator(s). This review is not an exception to that and so does not cover clinical aspects {however, seldom comments are made only if those issues are intimately / scientifically related & intermingle with ‘statistical aspects’ of the study}. Agreed that ‘statistical methods’ are used as just tools here, however, they are vital part of methodology [and so should be given due importance].* | So noted |
| *COMMENTS: Though the use of non-parametric Spearman’s ‘Correlation coefficient (ρ)’ {instead of parametric ‘Pearson’s} is appreciated, as most of measures/tools used are [though appropriate] yield data that are in ‘ordinal’ level of measurement and then application of suitable non-parametric test(s) is/are indicated/advisable even if distribution may be ‘Gaussian’ (i.e., normal)]. Moreover, most of the ‘Spearman correlation Coefficients’ reported in Table 3 [Spearman correlations of screening criteria by disclosed to MECSH nurse] are numerically very small {though sometimes significant}. In this context, please read the following:*  *Statistical test usually used to assess significance of Pearson’s ‘Correlation coefficient (r)’ is ‘t’ [where t = { r √ [(n-2) / (1-r^2^)] }for df=n-2, n is sample size] and here Ho is that the population/standard value of ‘r’ is zero. You need r=0.878 to be significant at 5% when n=5 but you need r=0.273 only, if n=50 & you need r=0.088 only, if n=500. Because ‘P-value’ heavily depends on sample size, it is customary to use the (available in most text books on ‘Biostatistics’ or on ‘www/net’) guidelines very strongly suggesting] to consider an absolute value of ‘Correlation coefficient for interpreting positive or negative correlations (and do not rely only on corresponding ‘P’-value but also consider an absolute value of ‘Correlation coefficient’). [This argument is equally applicable to* *non-parametric* *Spearman’s ‘Correlation coefficient (ρ)’ as well.]*  *This is pasted from one standard textbook on ‘Research Methodology’ and I am sure that the authors already know these things, however, it is very essential to keep the limitations in mind while interpreting results.* | We agree the absolute value of the correlation is considered as well as its statistical significance and thus had presented the correlations values in the results table 3 (not the p-values). We have submitted table 3 now as Figure 1.  Further, we directly acknowledge the limitation of the results in the text on line 200 (original manuscript) which states that the correlations reported are weak (<0.3). |
| *In fact, I wonder “how even ‘Spearman’s Correlation coefficients (ρ)s’ are calculated as most of the variables are (seems to be as per lines 167-8: Spearman correlations were conducted to test the association between the presence/absence of screening factors and presence/absence of disclosed ACE risks.) ‘binary/dichotomous’ [where only biserial or point biserial correlation coefficients are appropriate”. Will you please explain? (may be correctly done, but explanation is essential).* | After consideration we agree that this is not the best measure to report for this analysis, and it would be more appropriate to report the Phi correlation. After re-checking the results, we have updated table 3 to present these coefficients. NOTE: We would like to point out, that in a dichotomous vs dichotomous assessment of correlation, Pearson’s r, Spearman’s Rho, and Phi all produce the same results (see screen shots of SPSS output below) and as such no values reported in table 3 have changed. Note we converted table 3 into a figure following the recommendation in the table formatting rules “the safest solution is to make it into a figure”. |
| *Major part (lines 309-317: In this population, maternal smoking in pregnancy could be considered to be acting as a proxy for substance misuse, involvement with child protection services, mental health issues and family violence, suggesting that in similar populations particular consideration be given to additional and sustained support for mothers who are smoking in pregnancy. Not living with another adult was a proxy for unstable and violent relationships and major mental health issues; and anxious mood in the weeks prior to screening in pregnancy as a proxy for anxiety/depression and mental health issues during the subsequent two years. Maternal age was not a useful indicator of risk, however, receipt of state benefits was a proxy for a complex family environment) of ‘conclusion’ section is not really a [rather may/cannot be the] part of conclusion. Opinion(s) [may be even if learned from this experience], in my opinion, are not to be included in ‘conclusions’ of the study.* | We agree that the term ‘proxy’ is not helpful here, and have rewritten the conclusion to more appropriately reflect the study results. |
| *Overall, the presentation is confusing with respect few points {example – lines 139-140: 363 women were allocated to the trial intervention arm, and 352 commenced the intervention program what is the relevance of such grouping? No group comparison is clearly seen anywhere later. How the ‘allocation’ was performed is not given.* | We accept that reference to the parent study might be confusing to the reader. To help clarify this a reference to the parent study has been included in the Sample paragraph. We also have added text throughout the methods section to make clearer that this sub-study was only with those randomly allocated to the intervention arm of the parent trial. |
| *Moreover, at few places {example - lines 167-8: Spearman correlations were conducted to test the association between the presence/absence of screening factors and presence/absence of disclosed ACE risks as we never conduct the correlations} construction of a sentence needs to be checked, I guess.* | This sentence has been reworded. It now reads: Phi correlation coefficients and associated significance levels were calculated to measure the association between the presence/absence of screening factors and presence/absence of disclosed ACE risks. |
| *Limitations of the study are not discussed in a separate section (expected). Lines 295-97 mention only one limitation [These results cannot be generalized to population prevalence as the data were limited to those women who disclosed two or more screened-for sociodemographic factors in the context of recruitment for a sustained nurse home visiting trial.] though, which is not enough. {Does that mean {according to authors} there are none?} In my opinion, though it is a straight-forward simple study (not many methodological issues), contributes a marginal/minimal.* | We have added a heading ‘Limitations’. It should be noted that in addition to the lack of generalization, the limitation of the method of gathering the disclosure data was included in the original manuscript. Nonetheless, the limitations section has been expanded to again note the smallness of the correlations and that the disclosures were not verified by the researchers. |
| *As pointed out in ‘important note’ above “This review pertains only to ‘statistical aspects’ of the study and so ‘clinical aspects’ should be assessed separately/independently. In my opinion, to rescue this article (which is difficult but not impossible), lot of re-vision is needed. Therefore, just short of ‘rejection’, I am recommending “Major Revision”.* | Thank you for the opportunity to make these revisions. |

| REVIEWER #2 |  |
| --- | --- |
| *The purpose of this manuscript was to examine the association between sociodemographic measures collected at entry to a home visiting program and risks were identified based on disclosures to the nurse and measured in subsequent nurse case files. Screening was reported by the mother at the antenatal clinic and risks were identified by nurses through a retrospective case audit following HV services up to age 2 years of the child. This unique dataset explores an important topic in the field. However, I came away with totally different interpretation of the data presented. Specifically, the very weak associations (although statistically significant) between screening measures and subsequent risks imply that these are not good measures from a sensitivity/specificity perspective in identifying families who may benefit from sustained home visiting.* | We have noted the small correlations in the original manuscript, and further added this to the limitations section and conclusion. Our intent in this study was to record the prevalence of disclosed ACE risks (now made clearer in methods section) and to explore patterns of disclosed risk associated with sociodemographic risks noted in pregnancy rather than to test the sensitivity or specificity of the screening measures, particularly given that the risk disclosures were not objectively assessed (as now more strongly noted in the limitations section). As also noted in the limitations section, the intervention may have mitigated the risks. Consequently, the method would not support conclusions about the sensitivity/specificity of the screening. |
| *The introduction overall is well-written and clear. The authors capture the main issues the field is grappling with. The authors make a compelling case that a non-sensitive screening tool with high specificity would be ideal for many reasons. I also suggest the authors cite work in the US that utilized data from birth certificates that are highly predictive of later child maltreatment. However, I am not sure that the items in the screening tool presented here are actually non-sensitive since they include socially stigmatized domains such as poverty, maternal smoking, and mental health concerns that at least in most “Western” contexts would be subject social desirability.* | Thank you for directing us to the US studies utilizing birth certificate data. We have reviewed these studies, and included that by Buek et al 2019 in the introduction. We agree that some of the screening tool items may be considered sensitive, however, the cited screening tool study (original submission reference 23) indicated that the tool was acceptable to the women completing it in the public spaces, as noted in the manuscript. |
| *There is another limitation to the approach that is not considered, it appears that the ACEs are risks disclosed to the nurse, while the screening tool is self-reported by the mother. It is not clear whether these disclosures had to be reported directly from the mother to the nurse, or if the nurse could observe a risk and this counts as a disclosure. For example, if a nurse knew that a family was involved with CPS but the mother never disclosed this detail, how would the nurse document this in the spreadsheet? We then wade into territory regarding subjective/objective assessments and agreement between different reporters. But this is a fairly minor issue.* | We agree, and have noted limitations regarding the subjective/objectiveness of the disclosures in the limitations section. |
| *The major concern regards the “weak” correlations, as stated by the authors. In the conclusions the authors provide an interpretation that since the association between smoking and substance misuse was .273, that this is “particularly correlated”. I would disagree. Similar the correlated between anxious mood and anxiety was .186, this is quite a weak association. It is then suggested that these are now “proxies” for actual risks which to me is a very dangerous conclusion. Although the stakes in false-positives or negatives for this type of prediction are low (worst case you get offered a home visiting program), we should not equate a low association with a valid predictor.* | As noted above in response to Reviewer #1, we have removed the language of ‘proxy’, and noted the weakness of the correlations in the limitations and conclusion as well as the results. |
| *I was also curious why multivariable models were not used to see how screening measures predicted risks, while controlling for all of these factors, but perhaps this was not the goal of the research.* | As noted above, the goal of the research was not to verify, or create models for risk prediction, particularly given the method of the disclosures and potential impact of the intervention. |

## SPSS ouput

NONPAR CORR

/VARIABLES=riskfactor_age A_SubMisuse_d

/PRINT=SPEARMAN TWOTAIL NOSIG

/MISSING=PAIRWISE.

Nonparametric Correlations

CROSSTABS

/TABLES=riskfactor_age BY A_SubMisuse_d

/FORMAT=AVALUE TABLES

/STATISTICS=CHISQ PHI CORR BTAU

/CELLS=COUNT

/COUNT ROUND CELL.

Crosstabs
