# Supplementary material for: Identification of families in need of support: Correlates of adverse childhood experiences in the right@home sustained nurse home visiting program
Source: PLoS One. 2022 Oct 3;17(10):e0275423. doi: 10.1371/journal.pone.0275423 (PMC9529103; doi:10.1371/journal.pone.0275423)
Supplement: Attachment — Submitted filename: Response to Reviewers.docx [file pone.0275423.s004.docx]

| Requirement | Response |
| --- | --- |
| Specifically, we require that conclusions are presented in an appropriate fashion and are supported by the data. We still have concerns about some of the statements in your conclusion. For instance, you indicate that "Smoking in pregnancy was particularly correlated with substance misuse" (line 256). We would suggest removing "particularly" in this case as only weak correlation is reported and we feel your data does not support the conclusion here.    In addition, you indicate that "Sociodemographic screening in pregnancy using non-sensitive questions in this study appropriately identified a population of families with risks for ACEs, for whom sustained home visiting support may be of benefit to mediate the impact of risk and improve outcomes." (line 313-315). We do not feel this is supported by your data -  only (weakly) correlation was presented for the sociodemographic characteristics with ACEs, and that does not "identify" population of families with risks for ACEs.    Please ensure that all the statements in Conclusion are supported by your data presented and not overstate your conclusions. | The word “particularly” has been removed.  The conclusion has been revised to be more circumspect in its conclusion. In particular the core part of the conclusion has been reworded to “Children’s exposure to ACEs has been recognized by WHO as a significant public health issue. This study showed a high prevalence of ACE risks in the screened population who received the right@home visiting intervention and demonstrated small, albeit significant patterns of correlations between individual sociodemographic screening factors and disclosed risks. Sociodemographic screening in pregnancy was undertaken using non-sensitive questions in this studythat women were comfortable to answer in a public waiting room and identified this high risk population.” and the statement “appropriately identified a population of families with risks for ACEs, for whom sustained home visiting support may be of benefit to mediate the impact of risk and improve outcomes” has been deleted.  The words “Although the correlations were significant but weak” have been added to the final sentence of the conclusion.  We believe that the conclusion now reflects the findings. |
| Ensure your manuscript meets PLOS ONE’s style requirements | We have checked the style templates and believe all documents are correctly formatted. |
| Funding Information and Financial Disclosure do not match | The following statement is correct: “This work is supported by the Victorian Department of Education and Early Childhood Development, the Tasmanian Department of Health, the Ian Potter Foundation, Sabemo Trust, Sidney Myer Fund, the Vincent Fairfax Family Foundation, and the National Health and Medical Research Council (NHMRC, 1079418). The funders had no role in study design, data collection and analysis, decision to publish, or preparation of the manuscript.  These funders have been added in the revised submission. |
| Competing interests | The authors have declared that no competing interests exist and this has been noted in our cover letter. |
| Data availability statement | We have stated that data are available upon request, as the data reported in this study contain sensitive participant information which are restricted to use for research purposes only and therefore cannot be made publicly available. This restriction is in accordance with our Participant Information and Consent Form and approved study protocol, governed by the Royal Children’s Hospital Human Research Ethics Committee (HREC 32296).  We have responded with this information in our cover letter and updated our Data Availability statement. We provide details as to how researchers can request access to the data via the Melbourne Children’s Campus data access platform LifeCourse or the Royal Children’s Hospital Human Research Ethics Committee. We note that this response has been accepted for a recently published PONE manuscript that drew on the same right@home dataset, written by some of the same authors as this submission (Bryson H, Mensah F, Price A…Goldfeld S. (2021) Clinical, financial and social impacts of COVID-19 and their associations with mental health for mothers and children experiencing adversity in Australia. PLOS ONE 16(9): e0257357. <https://doi.org/10.1371/journal.pone.0257357>) |
| Ethics statement in methods section | An additional section heading has been removed, and the ethics statement now appears as a subsection of Methods. |
| Reference list | We have checked the reference list to be complete and correct. |
| Review #1 notes all comments have been addressed | We note that the reviewer is satisfied that all their comments have been addressed. We note the reviewers comment about the statistical test used, and are satisfied that the most appropriate tests have been used. We have also reviewed that complementarity of the figures in the text and the tables and are also satisfied that the text and the tables present the analysis clearly. |
